# Supplementary material for: CNApp, a tool for the quantification of copy number alterations and integrative analysis revealing clinical implications
Source: eLife. 2020 Jan 15;9:e50267. doi: 10.7554/eLife.50267 (PMC7010409; doi:10.7554/eLife.50267)
Supplement: Transparent reporting form [file elife-50267-transrepform.docx]

***eLife’s* transparent reporting form**

We encourage authors to provide detailed information *within their submission* to facilitate the interpretation and replication of experiments. Authors can upload supporting documentation to indicate the use of appropriate reporting guidelines for health-related research (see [EQUATOR Network](http://www.equator-network.org/%20)), life science research (see the [BioSharing Information Resource](https://biosharing.org/)), or the [ARRIVE guidelines](http://www.plosbiology.org/article/info:doi/10.1371/journal.pbio.1000412) for reporting work involving animal research. Where applicable, authors should refer to any relevant reporting standards documents in this form.

If you have any questions, please consult our Journal Policies and/or contact us: [editorial@elifesciences.org](mailto:editorial@elifesciences.org).

**Sample-size estimation**

- You should state whether an appropriate sample size was computed when the study was being designed
- You should state the statistical method of sample size computation and any required assumptions
- If no explicit power analysis was used, you should describe how you decided what sample (replicate) size (number) to use

Please outline where this information can be found within the submission (e.g., sections or figure legends), or explain why this information doesn’t apply to your submission:

We have submitted a *Tools and Resource* manuscript presenting our web-tool CNApp, a shiny tool to perform a quantification of copy number alterations and an integrative analysis with clinical and molecular annotated data.

To test its applicability and performance, we used publicly available segmented data from The Cancer Genome Atlas (TCGA) in three different approaches: (i) 10,635 samples spanning 33 cancer types from TCGA pan-cancer dataset to quantify the copy number alteration burden; (ii) 370 Liver Cancer-Hepatocellular Carcinoma (LIHC) samples to identify the most recurrently affected genomic regions; and (iii) 309 samples from the Colon Adenocarcinoma (COAD) dataset for which the colorectal cancer consensus molecular subtype (CMS) was known to test the ability of CNApp as a classification tool.

**Replicates**

- You should report how often each experiment was performed
- You should include a definition of biological versus technical replication
- The data obtained should be provided and sufficient information should be provided to indicate the number of independent biological and/or technical replicates
- If you encountered any outliers, you should describe how these were handled
- Criteria for exclusion/inclusion of data should be clearly stated
- High-throughput sequence data should be uploaded before submission, with a private link for reviewers provided (these are available from both GEO and ArrayExpress)

Please outline where this information can be found within the submission (e.g., sections or figure legends), or explain why this information doesn’t apply to your submission:

Due to the large number of samples tested in our analysis, no replication sample sets have been used to perform the genomic analysis.

**Statistical reporting**

- Statistical analysis methods should be described and justified
- Raw data should be presented in figures whenever informative to do so (typically when N per group is less than 10)
- For each experiment, you should identify the statistical tests used, exact values of N, definitions of center, methods of multiple test correction, and dispersion and precision measures (e.g., mean, median, SD, SEM, confidence intervals; and, for the major substantive results, a measure of effect size (e.g., Pearson's r, Cohen's d)
- Report exact p-values wherever possible alongside the summary statistics and 95% confidence intervals. These should be reported for all key questions and not only when the p-value is less than 0.05.

Please outline where this information can be found within the submission (e.g., sections or figure legends), or explain why this information doesn’t apply to your submission:

This information can be found either in the Methods and in the corresponding figure legends.

(For large datasets, or papers with a very large number of statistical tests, you may upload a single table file with tests, Ns, etc., with reference to sections in the manuscript.)

**Group allocation**

- Indicate how samples were allocated into experimental groups (in the case of clinical studies, please specify allocation to treatment method); if randomization was used, please also state if restricted randomization was applied
- Indicate if masking was used during group allocation, data collection and/or data analysis

Please outline where this information can be found within the submission (e.g., sections or figure legends), or explain why this information doesn’t apply to your submission:

All clinical and molecular variables corresponding to samples analyzed in our manuscript were retrieved from the TCGA project by using the *TCGAbiolinks* R package. CMS and MSS/MSI information for the colon cancer sample set was provided by Dr. Rodrigo Dienstmann from Vall d'Hebron Institute of Oncology (Barcelona, Spain), to whom we would like to express our gratitude.

**Additional data files (“source data”)**

- We encourage you to upload relevant additional data files, such as numerical data that are represented as a graph in a figure, or as a summary table
- Where provided, these should be in the most useful format, and they can be uploaded as “Source data” files linked to a main figure or table
- Include model definition files including the full list of parameters used
- Include code used for data analysis (e.g., R, MatLab)
- Avoid stating that data files are “available upon request”

Please indicate the figures or tables for which source data files have been provided:

Additional files to run our CNApp tool and the R source code can be found at GitHub (https://github.com/ait5/CNApp).
